# Oxidative-stress-induced telomere instability drives T cell dysfunction in cancer

**DOI:** 10.1016/j.immuni.2026.02.015

**Published:** 2026-02-26

**Authors:** Dayana B. Rivadeneira, Sanjana Thosar, Kevin Quann, William G. Gunn, Victoria G. Dean, Bingxian Xie, Angelina Parise, Andrew C. McGovern, Kellie Spahr, Konstantinos Lontos, Ryan P. Barnes, Marcel P. Bruchez, Patricia L. Opresko, Greg M. Delgoffe

We recently became aware of an error in Figure 2. We noticed that the representative histograms in Figures 2D and 2G were duplicated during final figure formatting. During figure production, we inadvertently copied the DCFDA staining histogram from the analysis software for both Figures 2G and 2D. We have fixed this error by adding the correct representative histogram for CD39 expression in Figure 2G. The data points used for the tabulated analysis are not affected by this formatting error. Further, the conclusions demonstrated in this work are similarly unaffected. The authors apologize for the error and any inconvenience it may have caused.

## Figures and Tables

**Figure 2.**
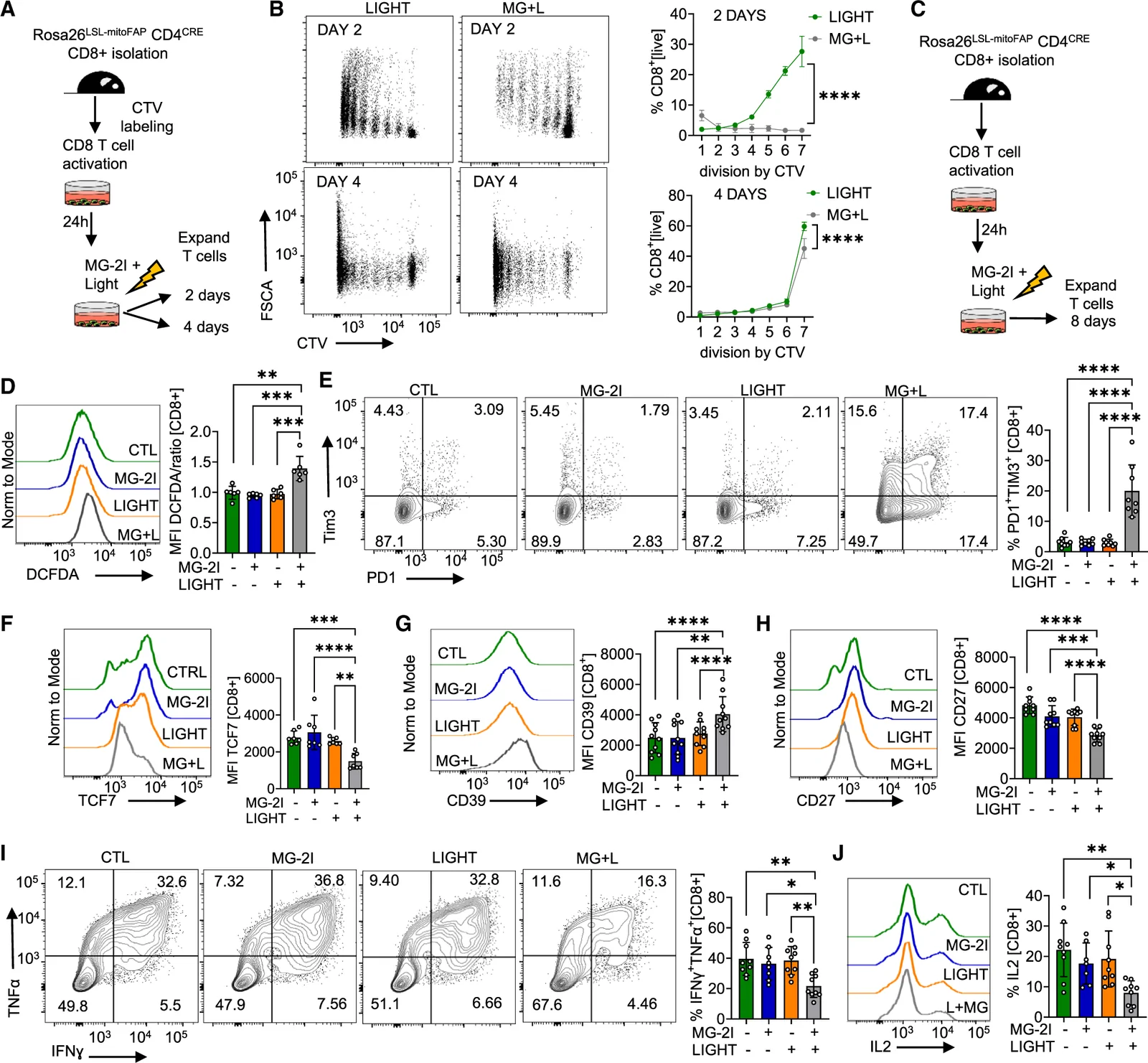
Mitochondrial oxidative damage induces T cell dysfunction (corrected)

**Figure 2.**
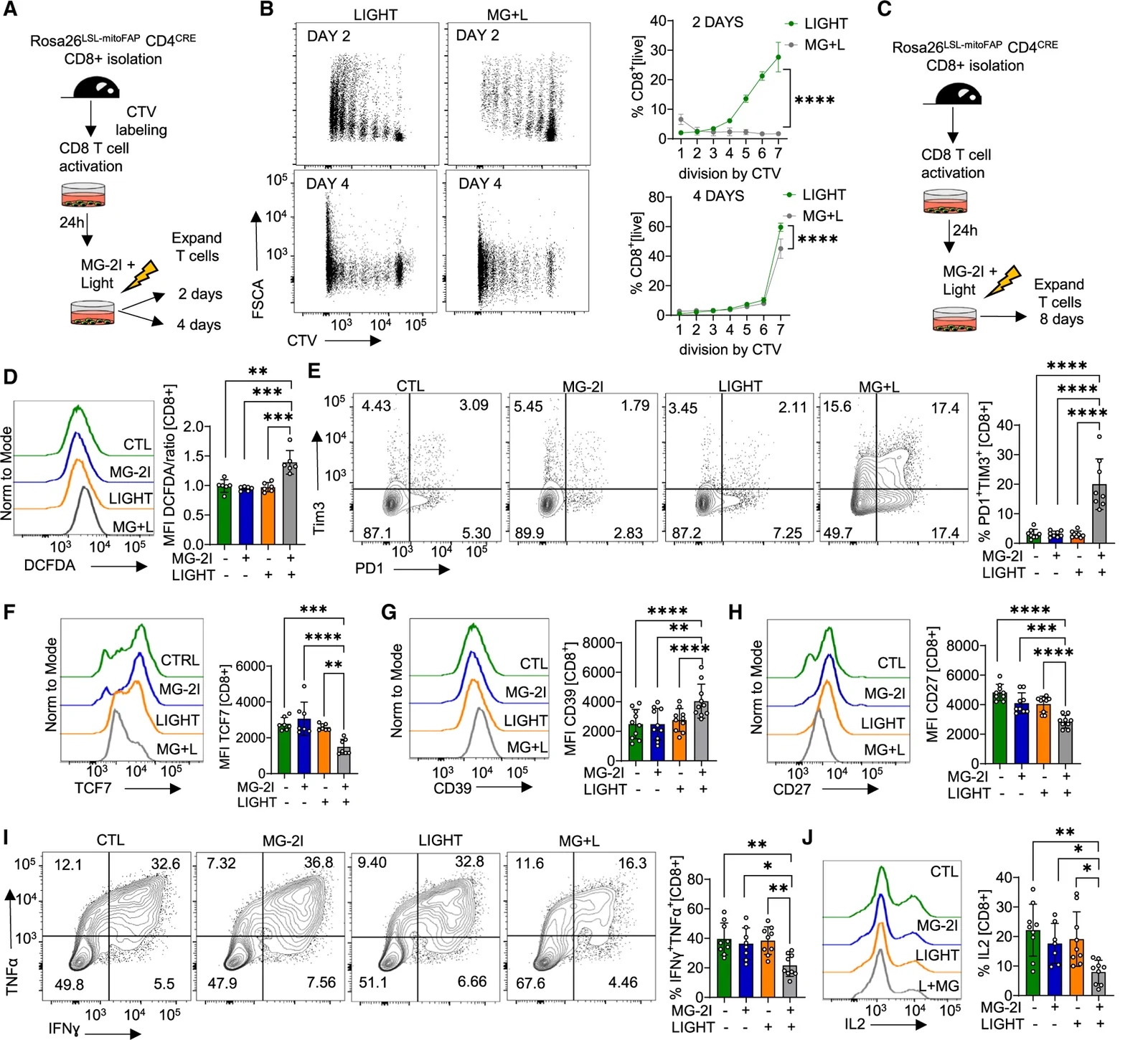
Mitochondrial oxidative damage induces T cell dysfunction (original)

